# Individualism and Collectivism at Work in an Era of Deindustrialization: Work Narratives of Food Delivery Couriers in the Platform Economy

**DOI:** 10.3389/fsoc.2020.00049

**Published:** 2020-07-28

**Authors:** Paul Stewart, Genevieve Shanahan, Mark Smith

**Affiliations:** People, Organisations & Society, Grenoble Ecole de Management, Grenoble, France

**Keywords:** platform economy, digital capitalism, deindustrialisation, gig workers, individualism-collectivism, collective worker, collectivism

## Abstract

Supposedly emblematic of digital capitalism, the rise of the gig economy is frequently taken as a cipher for the developing deindustrialisation of western societies. It is tempting to interpret the shift of manufacturing jobs to the global south and their replacement with service sector jobs as a one-way street, leading to the demise of decent work and the rise of work characterized by precarity, low pay, low skill and a non-unionized workforce. However, the reality is inevitably more complex. In the first place, pessimism may be attributed to a rose-tinted view of the experience of former industrial employment in the global north resulting from a questionable assumption about the nature of the jobs that occupied most people in former industrial societies. Certainly, deindustrialisation is not leading to “de-working,” that is, working less for the same money. With respect to gig work, autonomy and flexibility are central to labor inducement and hence labor control. Yet at the same time, and linked to the latter, we need to explore another deep-rooted phenomenon: the persistence of work*space* collectivism. Our evidence derives from qualitative interviews with gig workers in the food delivery sector in a number of European countries. We highlight the extent to which couriers profess a variety of understandings of the character of platform economy labor processes. A range of narratives emerge including platform work as leisure, as economic opportunity, and as collectivist labor. Moreover, individuation, attendant upon the character of the physical labor process, did not lead in any straightforward way to individualism in social labor processes—contrary to our expectations, we in fact witnessed forms of collectivism. Collectivism is to be distinguished from “types of solidarity” described by Morgan and Pulignano (2020) whereby neo-liberalism has transformed a range of institutional forms of labor solidarities. By contrast, we are concerned with the persistence of the collective worker within the changing sociological structure of work. This echoes the earlier finding by Stephenson and Stewart (2001) that collectivism endures even when behaviourally absent and indeed even in the context of individualized working—termed “whispering shadow.” Thus, the objective of the paper is to explore the forms of actor individualism and collectivism identified in our research. Given platform apps' external control, the gig economy spatially separates workers while at the same time requiring cognition of colleagues' collective work and labor process. Notwithstanding structural processes separating workers-in-work, platforms also witness the instantiation of forms of collectivism. Deindustrialisation is neither the end to collectivism nor trade unionism. Rather than post-work, then, we explore the problematics of *plus* work and *variant* collectivisms.

## Introduction

Those who see a terminal crisis of labor movements tend to see the contemporary era as one that is *fundamentally new and unprecedented*, in which global economic processes have completely reshaped the working class and the terrain on which labor movements must operate. In contrast, those who expect the re-emergence of significant labor movements tend to perceive historical capitalism itself as being characterized by recurrent dynamics, including the continual re-creation of contradictions and conflict between labor and capital (Silver, 2003).

The rise of organizations operating in the so-called “gig,” “sharing” or “platform” economy has led to an increasing interest in the changing nature of work and working conditions for those employed in the sector (Berg, 2016). For an interesting discussion of defining vocabulary see, inter alia (Bughin et al., 2016; Huws, 2019; Morozov, 2019). ^1^ What is more, we must ask what the political economy of this sector tells us about the evolving character of work and employment in the global north. There has been a focus around the extent to which definitional ambiguity and contractual (in)security represent new challenges to workers and new dimensions of precariousness (Goudin, 2016). However, while concerns among academics and policy makers about the rise in precarious work have been visible, as have campaigns for greater security, these conditions appear to be transparent to the workers upon taking up such roles. Furthermore, there has also been defense of the flexibility and other benefits associated with such work, not only from the platforms but also from the workers themselves. This paper explores the narratives around the tensions and contested expectations of platform workers through a study of food delivery couriers. These narratives are significant in their articulation of dimensions of actor dissonance within the platform economy and moreover the persistence of collectivist attitudes in a society characterized by deindustrialization. Not only is collectivism present but the attitudes of platform workers reveal the spaces, character, and radical possibilities inherent in the social recomposition of the labor force in digital capitalism. Precarity, insecurity and individualism have always been a feature of capitalism albeit reflected in variant patterns of social solidarity and insolidarity. The discourses of individualism that we highlight are in fact no more fundamental to working class culture in digital capitalism than they were to workers in industrial capitalism.

In line with Stewart and Stanford (2017), we adopt the terminology of the platform economy whereby the use of technology to match workers to discreet tasks has created what may be considered a new paradigm of what work means and provides (see also Taylor, 2017). Platform workers are paid piecemeal for tasks completed and are frequently treated as self-employed contractors. Yet while they are deprived of the rights and benefits of employees, neither do they necessarily exhibit the same autonomy and freedoms of typical self-employed workers (Rosenblat and Stark, 2015; Schmid-Drüner, 2016). As Drahokoupil and Jepsen (2017) point out, platforms present the work they offer as distinct from traditional employment in various ways, evidenced in terms of the flexibility the worker enjoys, rooted in part in the identity of the supposedly typical platform worker—young, male, fit, no dependents. This logic frequently is utilized to justify the platforms' non-compliance with the traditional, legal requirements of employers, especially regarding income, and job security. Moreover, flexibility and autonomy are presented as simply incompatible with the “traditional” employment relationship. Thus, according to this understanding, for firms operating platform commodity and labor utilization strategies, their non-compliance is not a case of worker exploitation but rather a requirement of the freedom desired by platform workers themselves.

In spite of this professed “freedom” in the platform context, a series of obligations and expectations emerge, both codified and non-codified. The divergence of expectations between (some) workers and platforms led us to investigate why some workers appear satisfied with the work, while others become disgruntled over time or take on the work grudgingly from the start. Moreover, the implications of worker expectations resulting from new patterns of capital accumulation and their attendant workplace and work space social solidarities are therefore pertinent to our research. A number of different theoretical approaches can help make sense of these expectations, including employment relationships (Marsden, 1999), social exchange theory (Cropanzano and Mitchell, 2005) and critical labor process theory (Martínez Lucio and Stewart, 1997; Stephenson and Stewart, 2001; Vidal, 2007; Benanav, 2019; Casilli, 2019; Morozov, 2019; Tassinari and Maccarrone, 2019). In our research we explore how workers interpret met and unmet expectations through exploratory semi-structured interviews with couriers working for food delivery platforms in European countries—specifically the UK, France, Italy and Germany. Our analysis points to a series of competing narratives among couriers as they seek to make sense of their multiple identities.

The competing explanations offered by couriers have parallels with Pasquale (2016) “Two Narratives of Platform Capitalism,” in which he highlights the existence of a dominant narrative and counter-narrative regarding the platform economy. On the one hand, the neoliberal economy account tells “a simple narrative about the incentives created by reducing transaction costs and creating more opportunities for individuals and firms to compete to provide services” (Pasquale, 2016). A progressive counter-narrative highlights the ways in which platforms dominate through the individualization of risk and the use of capital to lobby against regulation as much as through fair competition. In this respect, we argue that the counter-narrative itself constitutes an important basis to the reconstitution of the collective worker in late capitalism (Jameson, 1984) in the context of the seeming individualization of the labor process. While for management the latter appears to provide a structural impediment to labor collectivism, we argue that on the contrary it can be seen also to highlight the persistence of the collective worker—a worker who is nevertheless displaced, distanciated and yet integral to the very structural forces that were supposed to end social solidarities at work (Woodcock, 2017; Tassinari and Maccarrone, 2019).

This paper is structured around six sections. Following this introduction, the next section explores the state of research on the platform economy. We then explore different theoretical approaches that shed light on the process by which expectations may be shaped and broken. Expectations are important in this regard since they are crucial to the motivational character necessary in all affective relationships and specifically the wellspring of actor behavior including individualistic and collectivistic tropes (and a mixture of both). We next outline our strategy for data collection. The final substantive section uses this data to explore the competing narratives of the food delivery couriers in relation to their expectations, obligations, and contractual status. We conclude by assessing the import of worker narratives in making sense of the reconstitution of the collective worker in the platform economy. In so doing we suggest that while patterns of (re)collectivism are inherently part of the capitalist labor process, the shape of collectivism is always tied to dissonant discourses. This means that just as worker collectivism does not go away and is continually reconstituted by new forms of capital accumulation (see Silver, 2003), so too are reconstituted forms of accommodation to precisely these patterns of accumulation. As our interviews indeed highlight, individualism is reflected in the persistence and recreation of the idea of the worker as a free (neoliberal) agent. The latter thus constitutes a tension with the collectivist, qua solidaristic, narratives also expressed by our interviewees. That said, and echoing Stephenson and Stewart (2001), collectivism is reducible neither to trade unionism nor anti-management solidarity. This is another way of saying that, while labor agency remakes worker collectivism, it also remakes individualism-individualization, both critical to the fate of the collective worker (Martínez Lucio and Stewart, 1997; Tassinari and Maccarrone, 2019). In making sense of worker agency, including our concern with the tension between individual and collective discourses, we focus on the idea of worker expectations from the standpoints of employment relations including a labor process perspective while also paying due attention to other theoretical perspectives. However, prior to this we turn our attention to a range of literature exploring the distinctive character of platform working that, while important, can be said to largely eschew discussion of the impact of new patterns of working on employees' individual-collective identities.

## The Platform Economy

In spite of the relatively recent rise of digital platforms, there is already an emerging body of research on the nature of work created by such technologies. Initially conceived as the “sharing” economy (see fn 1 above; Cohen and Sundararajan, 2015; Sundararajan, 2015; Codagnone et al., 2016) it has become apparent that the collective and not-for-profit origins of such organizations have been superseded to a greater or lesser extent by more conventional concerns around the power of large organizations (Balaram, 2016; Scholz, 2016), tax and regulatory avoidance (Minter, 2017), and the exploitation of labor (Howcroft and Bergvall-Kåreborn, 2014). The latter has spurred interest in the risks of increasing precariousness (Goudin, 2016), the needs for new forms of special protection, such as basic income (Standing, 2011), and regulatory categorization (Schmid-Drüner, 2016). Moreover, an associated literature has raised questions regarding the relationship between organizational form and labor utilization strategies, including exploitation, and hence labor responses within and beyond organized labor (Drahokoupil and Jepsen, 2017).

However, reflecting the emerging nature of the field, there is no consensus on a definition of work in this part of the economy, such that some conceive it more broadly than others do. As a result, a variety of terminologies have emerged to refer to the same or related phenomena, including “gig work” (Stanford, 2017), “independent work” (Bughin et al., 2016), “on-demand work” (Berg, 2016), and “crowd work” (Felstiner, 2011). Woodcock and Graham (2019), Huws (2019), and Benanav (2019) consider all of these definitions from a macro-sociological standpoint. The gray literature, by contrast, tends to focus especially upon individual features as being fundamental to platform work. For instance, the Chartered Instituted of Personnel and Development (CIPD, 2017) defines platform work as “a way of working that is based on people having temporary jobs or doing separate pieces of work, each paid separately, rather than working for an employer” (2017, p. 4), while the RSA emphasizes the use of apps to sell one's labor (Taylor, 2017, p. 25).

Much literature focuses on the question of defining platform work and its various subsets (see, inter alia, Wood et al., 2018a,b). Codagnone et al. (2016) offer a useful distinction between Online Labor Markets (OLMs) and Mobile Labor Markets (MLMs). While the former involves work delivered purely online, such as micro tasking through platforms like Amazon Mechanical Turk, as well as larger projects that are more skilled commissioned through platforms such as Upwork (Wood et al., 2018a). The latter, by contrast, involves a physical, and therefore local, presence and includes meal delivery tasks that are the focus of this paper.

Aside from digital mediation, the features of precariousness, blurred boundaries, and opaque contracts are not novel. Task-based, informal and irregular work has always existed in various forms, including childminding, day labor, contracted construction work, etc. (Peck and Theodore, 1998). Alkhatib et al. (2017) and Stanford (2017), for example, both compare modern on-demand labor to historical examples of piecework. However, platform technology permits a high level of commodification of labor. De Stefano highlights the connection between the pervasive mediation of work via apps and software and the risk that the workers themselves are “identified as an extension of an IT device or online platform” (2016, p. 5). This in turn can lead to associated expectations that workers should perform as seamlessly as a machine, followed by disproportionately negative reactions when they cannot. The euphemisms of the platform economy—“gigs,” “tasks,” “rides,” “pin money”—suggest that this work is not significant enough to require ordinary labor protections (Codagnone et al., 2016; De Stefano, 2016). Yet research suggests platform work is performed by a diverse range of people, including those who treat it as “a spare-time activity” (Eurofound, 2015, p. 113) and those for whom it is the primary source of income (Berg, 2016).

It is again possible to draw parallels with more traditional forms of precarious work that do not necessarily rely on the technology of platform work. De Stefano identifies platform work as “part of broader phenomena such as casualization and informalisation of work” (2016, p. iii). This erosion of the traditional employment relationship dates back several decades and cannot be attributed solely to the rise of platform-mediated work (Codagnone et al., 2016). A common feature across these precarious types of work—platform and non-platform—is the shifting of risk from the firm to the worker (Friedman, 2014; De Stefano, 2016). Indeed, Howcroft and Bergvall-Kåreborn (2014, p. 213) underline this risk in relation to OLMs, which can mean that:

“functions once performed by internal employees can be outsourced to an undefined pool of digital labor using a virtual network. This enables firms to shift costs and offload risk as they access a flexible, scalable workforce that sits outside the traditional boundaries of labor laws and regulations.”

Casualization is not new and these risks for workers are reflected in the debates surrounding the contractual status of platform workers. Schmid-Drüner considers recent literature and rulings regarding the legal status of platform workers, noting that while, “[i]n most cases, platform workers are classified as self-employed, […i]t is contested that the regular classification of platform workers as ‘self-employed' really does justice to the original idea of self-employed” (2016, p. 5). By using the European Working Conditions Survey (EWCS), Oostveen et al. (2013, p. 1) identify “economically dependent workers,” that is, workers who are registered as self-employed but resemble employees in important ways. For instance, they may “depend on a single employer for their income and thus have no real autonomy in running their ‘business,”' suggesting that some of these workers tend to be more like employees in terms of control of time, tasks and place of work. Exploring perspectives addressing changes in labor market structure, such as those suggested by, inter alia, Rubery et al. (2018) may be more useful in getting to grips with the way researchers can consider gig work not only in terms of its insecure precarious nature. The issue is also that of the relationship between the state (viz, labor market protections qua de-commodification measures), the labor market and worker activity.

Finally, perhaps unsurprisingly, extant research tends to focus more on the consequences for those working in the platform economy in terms of their contractual status and working conditions as opposed to exploring the consequences of the platform economy for worker individual and collective consciousness. Taking this as a point of departure, we focus on the expectations of those working in the platform economy, including the significance of these expectations for notions of individualist-collectivist perceptions of actor identity.

## Making Sense of Worker Expectations—Not all About Individualization

In considering the expectations of workers in the platform economy, a number of theoretical perspectives become apparent, including employment relationships (Marsden, 1999, 2000 and see reference to Rubery et al., 2018 above), social exchange theory (Cropanzano and Mitchell, 2005) and critical labor process theory (Martínez Lucio and Stewart, 1997; Gandini, 2018; Tassinari and Maccarrone, 2019). While recognizing the relevance of psychological contract theory (Cullinane and Dundon, 2006), it has less relevance where our concern is with the structural fate and contextual tension created by tied discourses of individualism and collectivism in an era of deindustrialization. We consider each in turn.

For the employment relationship, the contract of employment is only partial since labor cannot be considered a commodity in the strict sense. Both employer and worker have an interest in the relation evolving, even to a limited degree (Marsden, 1999). The ongoing nature of the employment relationship provides employees with an assured continuity of income and the ability to plan for future events, while employers can rely on a regular labor supply that a strictly transactional relationship would put into question. Equally, employees benefit from income in non-work time such as holidays, leave and periods of illness, which underlines how employment relationships are highly embedded in institutionalized systems of welfare and protection whether provided by state or employer (Esping-Andersen, 1999).

The self-employed, or perhaps pseudo self-employed in the case of food couriers, may be categorized as having strictly transactional contracts, yet authors such as Wilkens and Nermerich (2011) and Marsden (2004) nevertheless point to relational aspects of their “contracts.” Self-employed workers' security is often rooted in their ability to move from one short-term project to another, progressively building a favorable reputation, rather than having that security provided by a specific employer. As a result, self-employed workers can value skill-development and networking in the projects they take on, and this can form the basis of a fair exchange: “As long as a job episode […] provides some opportunities for the future it is accepted as a reciprocal exchange relationship even though benefits are expected in future and not in present. Actual work relationships are evaluated in the light of opportunities for future development” (Wilkens and Nermerich, 2011, p. 79). Rubery et al. (2018) make a key intervention on this theme of what might be termed “job ownership-control.” They assess the nature of precarious work not in terms of its sui generis character but rather with respect to commodification-decommodification (i.e., economic protections-non-protections) of state-labor market relationships in six European countries. These relationships ultimately concern the extent to which state-labor market policies are orientated toward protecting workers when they are not in work. Thus, questions around job security and job control, over-determined by the pattern of the state's relation to the labor market, are societally specific and as such fundamentally crucial in making sense of the contours of job precarity. This is in part also the story about the search to define new forms of work in relation to the Standard Employment Relationship (SER) and while it does not define our research brief it is germane to our concern with gig working.

Social Exchange Theory (SET) provides another perspective from which to consider platform worker expectations. In their review of SET, Cropanzano and Mitchell (2005) identify that contexts where multiple exchange rules are employed simultaneously. This has been given less consideration in the literature, yet this is perhaps pertinent for couriers who experience overlapping and conflicting exchange logics with platforms, fellow couriers and clients. Competition is also perhaps a relevant consideration for riders who are in effect competing for “gigs,” but it is perhaps unlikely that they would go as far as to hurt another party for the relatively small benefits associated with most platform work. Nevertheless, the nature of unwritten trust-based relations in SET may provide some useful pointers to understanding the expectations of couriers working for platforms.

These approaches, while important in assessing the regulatory (viz. employment) context of worker behavior and engagement in the platform economy, are limited in accounting for the wider social structural context. Thus, we need to explore expectations and behavior more fully in relation to social and political power. This is because, in considering changes to employment in the gig (post-industrial) economy, exploring workers' views of labor control, exploitation, and solidarity allow for a closer assessment of the contrasts and continuities between different eras in work and employment. In this respect, we consider that the critical labor process perspective presents a more fruitful avenue for our research agenda since, in allowing for a critical assessment of the changing nature of power in the employment relationship in the (post-industrial) era of the platform economy, it sheds light on a double relationship. This may be characterized as a relationship between, on the one hand, worker exploitation and patterns of labor subordination-insubordination (the “how do workers struggle/not struggle” question), and on the other hand, the social and material context of that exploitation. The latter typically is understood as central to determining patterns, forms and orientations of subordination-insubordination (the collective worker question and its persistence through time). There is an archaeology to the so-called labor process debate which can be accessed elsewhere (Thompson and Smith, 2009). Since it is especially fissiparous we are concerned with one, albeit vital, aspect of the labor process that concerns the fate of the collective worker (Martínez Lucio and Stewart, 1997; Gall, 2020).

From our interviews we assess the variant strands in discourses reflecting the range of tensions we delineated above: the neoliberal and the counter-narratives. The counter-narrative, in particular, illustrates the social, and cultural contextual factors that block the translation of deindustrialization into decollectivism. Moreover, we see that the basis for a new collectivism is rooted precisely in the type of flexible work that was supposed to herald its demise. We thus argue that platform work itself creates the basis for a form of neo-collectivism. The accounts examined in this paper highlight the lack of a clear line of demarcation between employer control and market dictates, especially as platform algorithms become increasingly adept at reflecting and predicting market demand. We argue, therefore, that this constitutes a structural ambiguity in a worker's employment status partly determined by their degree of freedom in choosing when and how to work. Moreover, this ambivalence suggests conflict will be an inevitable part of this work paradigm. Deindustrialization is neither the end to collectivism nor trade unionism: rather than post-work we explore the problematics of *plus* work and *variant* collectivisms.

## Methods

One of the challenges in researching work in the platform economy is the socio-economic heterogeneity of platform workers. Platform work encompasses different sets of workers, from those who are high skilled (such as in high-level consulting) to low skilled (like data entry). To reduce this complexity, we focus here on one set of workers who have become a paradigmatic example of platform work in public debates: food delivery couriers, working for platforms such as Deliveroo, Uber Eats, Foodora, Just Eat, etc. Such workers have contested the definition of their employment status, given that these low-skill tasks are performed in the context of an ongoing relationship, even though platforms themselves publicly stress the lack of formal engagement. We used semi-structured interviews with 14 male platform workers (there are few women in the sector) between the ages of 20 and 36 across Europe (UK, France, Germany and Italy). The majority were students, five worked for delivery platforms as their primary job, and one was a more traditional freelancer, using platform work to top up his income (see Table 1). The limited sample size reflects the exploratory nature of our research and we complement with our analysis of protest materials from the four countries. Furthermore, the expert informants from Italy and Germany, a long with those from France and the UK, are used to complete the national protest materials in order to contextualize the responses of the interviewees within the public articulation of grievances at the societal level.

**Table 1:** Participant attributes.

**Age range**	**Experience level (months)**	**Financial dependence on platform**	**Platform**	**Status (PT: part-time; FT: full-time; SE: self-employed)**	**Vehicle**	**Int #**
**France**						
26–30	18	Dependent	1 and 2	PT, student, SE	Bike	FR1
20–25	3	Relatively dependent	2	PT, student, SE	Bike	FR2
20–25	<1	Relatively dependent	1	PT, student, SE	Bike	FR3
20–25	<1	Not dependent	2	PT, student, SE	Bike	FR4
20–25	1	Not dependent	2	PT, student, SE	Bike	FR5
20–25	10	Relatively dependent	1, then 2	PT, student, SE	Bike	FR6
26–30	18	Dependent	1 and 3	PT, student, SE	Bike	FR7
**UK**						
26–30	2	Dependent	1 and 2	FT, SE	Bike	UK1
20–25	4	Dependent	1	FT, SE	Bike	UK2
26–30	9	Dependent	1 and 2	FT, SE	Bike	UK3
36–40	10	Dependent	1, then 2 and 3	FT, SE	Car	UK4
31–35	18	Dependent	1 and 2	FT, SE	Bike	UK5
**Germany**						
20–25	6	Relatively dependent	1, then 2	PT, student, mini-job	Bike	DE1
**Italy**						
31–35	2	Not dependent	1	PT, traditional freelancer, SE	Bike	IT1

Systematic sampling of representative populations is made difficult by the specific characteristics of this workforce (Chai and Scully, 2019). We therefore approached couriers in informal gathering places, both physical and virtual, employing a convenience sampling strategy to access as many participants as possible in this dispersed and hard-to-reach population. We are mindful that the couriers who responded to our calls for participants are unlikely to be representative of the wider courier population, being both available and willing to participate in our study. However, in light of the existing research to date and our informal engagement with couriers' online support and protest groups, we are confident that the issues raised are consistent with the experiences of couriers more generally.

Platform couriers engaged in a wave of protests and strikes throughout 2017 across a number of European countries, and many of the actions were organized and publicized via social media. As part of our research, we conducted a systematic analysis of the complaints and demands associated with these actions to identify articulated worker interests in conflict with food delivery platform firms. As a first step, we constructed a timeline of significant strikes and demonstrations in the UK, France, Italy and Germany from news reports, beginning in August 2016. Other materials, including statements, leaflets, posters, placard images and banners, together with social media posts relating to the protests, were collected. These searches uncovered other lower-profile protests that enriched our data. Given that these groups tend to promote one another's efforts, we were able to identify additional groups claiming to speak for the couriers, thus this became an iterative process, with each group highlighting further events and groups. Once saturation was reached, the data were imported into NVIVO. We then reviewed the texts and images, with a focus on identifying concrete demands. While bearing in mind that these materials are not representative of all platform workers, we took the number of appearances of a given element to be a rough proxy for its importance at least to the protesting couriers.

Using these data we explore the relationship between what workers “expect” from their work and the importance of these expectations as drivers of individualism–collectivism. As Stephenson and Stewart (2001) argue, collectivism is reducible neither to worker behavior nor to worker expectations. Individualism-collectivism takes myriad forms premised as it upon the social-technical character of the work organization and the labor process. While platforms typically seek to foster individualistic attitudes qua transactional orientations amongst workers, in our research these were not exclusive of other orientations, including collectivist ones. Affirming transactional commitment did not exclude a collectivist orientation. After all, as writers from Goldthorpe (1968) to Huws (2019) have pointed out, feeling “cheated” or ignored by a firm promising positive working conditions (autonomy, independence, flexibility) has always been a great spur to solidaristic consciousness. As we discovered, a shake-up in employment experiences indeed proved to be a great driver of solidaristic consciousness and action.

## Findings

### Worker Expectations

Our initial analysis of the interview transcripts identified a number of emerging themes relating to expectations as workers in the platform economy. First, and in line with much of the existing research, there is an overriding concern regarding the status of couriers in relation to their contract of “employment,” often drawing comparisons with those working as employees in the sense recalled by Rubery et al. (2018) in their analysis of SERs. Second, we identify a series of obligations between couriers and platforms that the former perceived as having been transgressed. Third, we identify a series of exchanges between the couriers and other stakeholders—customers and restaurants.

As expected, given the ongoing debates and lack of legal clarity, few couriers classified their status as unambiguously that of either an employee or a self-employed worker. Couriers found themselves drawing parallels both with employees and more traditionally defined self-employed workers, while perceiving that they fell between the two:

“yeah, the way it works with [Platform 1] and [Platform 2] I guess is they're not actually employing me. I mean they are supplying the kit, with branding on it, but you don't actually—it even says it in the contracts that you don't have to wear the uniform and you are free to work for whoever you want, even if you are wearing our uniform. So I can do a [Platform 2] job covered in [Platform 1]. It doesn't matter, they don't really care about that. And I think it's to avoid them looking like they're employing me, I think it's a legal thing. But it helps me, it's great, I don't have to change my clothes or anything.” (UK1)

“In my situation I think it's a fair balance. But if I worked like all the day I think it's unfair because you must take an insurance and... it's quite “salariés déguisés” [hidden employees]—it's like if you work for someone but not officially” (FR3)

This was a source of frustration and perceived injustice for some:

“I felt like they had acted in a very disloyal way and had made it clear that they didn't value me as an employee. So they'd broken a sort of contract of honesty and reasonable behavior between us” (FR1)

In addition, beyond questions of employment status, interviewees reflected on expectations, their relationship with the platform, and moments when perceived obligations were not honored. Participants identified obligations they believed the platform had toward them:

“I think it's something maybe [Platform 2] should look at, try to look after the good hard worker. […] These kinds of companies should look at it cause these people would be very good workers. People who work really hard for them.” (UK4)

But also that these obligations were not always honored:

“When I signed up I was told that my wage would increase as time went on as a function of my behavior, my performance. So I worked very hard initially to ensure that I was in the upper percentile of people that'd get the extra money. And that was never, that never materialized, even though that was promised in writing to me, I have the emails.” (FR1)

Similarly, participants identified expectations the platform had of them:

“Personally, I think that we must be polite with the client because it's an image, and if you don't it can be a nuisance. It can be bad for the image for the enterprise, for [Platform 1], and for me.” (FR3)

“The first week that I was doing [Platform 1] after the training session, so after that they said ‘right, so, when are you going to work, we'd strongly encourage you to sign up for 3 or 4 shifts right now'. So I hadn't planned on doing that, I'd planned on taking a week and then signing up, but because of that I signed up for the weekend.” (FR1)

It also emerged that respondents considered relationships with other parties as important, including restaurants, customers and other couriers:

“It's not something that I should do or am obliged to do. It's a human thing that I did with him. […] I could have finished the delivery and told him ‘it's not my problem, call the support, they will call the restaurant'. […] you're not paid for it. […] 5 euros, I don't care, it's a human thing, and you can't be paid for a human thing.” (FR4)

“we do think about each other as well. like, anyone in that group if you're working for [Platform 1] and if you've got a problem or you're struggling everyone like helps and digs in and helps out that person because like I say it's just the way, it's like being part of a squadron I guess, you know you look after each other as a family in a way.” (UK1)

Through these emerging themes, we observe that platforms aim to foster transactional rather than relational relationships with their workers (Cullinane and Dundon, 2006). Such an approach supports their public and quasi-legal position that platform workers are not in fact employees but rather self-employed contractors. The rhetoric of these platforms has emphasized the lack of relational elements to their relationships with workers—for instance, by not requiring workers to act as brand ambassadors by wearing logoed uniforms, and explicitly confirming that couriers are free to simultaneously work for competitor platforms. The manner in which workers are paid, piecemeal for individual tasks, is perhaps the clearest public indication to workers that the relationship is purely transactional. There is no explicit system for directly rewarding anything extra couriers may do to advance the other interests of the firm. Couriers' multiple relationships, however, complicate the (straightforward) expectations between platform and courier and contribute to competing narratives, as discussed in the next section.

### Narratives of Individualism, Narratives of Collectivism

From their perspective, the couriers profess a variety of understandings of the courier-platform relationship, and express varying attitudes toward platforms and the platform economy. Following Pasquale, we reviewed the transcripts with a view to exploring the competing understandings in couriers' accounts of their work. We found that couriers employ at least three distinct narratives, threaded through our respondents' testimonies: platform work as leisure, as economic opportunity, and as collectivist labor. The first two roughly correspond to narratives put forward by platforms themselves, while the third reflects the account favored by dissatisfied couriers, as well as protestors and trade unions. Here we outline each of these three narratives, before exploring the implications for our concern with the relationship between individualism and collectivism in the courier labor process. In doing so, we identify a number of tensions between the three narratives (see Table 2).

**Table 2:** Narratives informing courier expectations.

**Platform work as:**	**Leisure**	**Economic opportunity**	**Collectivist labor**
Favored by	Platforms	Platforms	Unions/protestors
Nature of work	A sport (focus on physical performance) and/or a videogame (focus on app, tech)	Empowering, facilitating couriers' economic freedom	Menial, exploitative piecework, managed by algorithms
Courier identities	Students/young people	Freelancers/entrepreneurs/artists	Workers/victims with few options
Logic	Payment as side-benefit, pocket money	Individuals responsible for their own business, platform merely provides tools, at a cost	Platform claims profit comes from algorithm, but really generated by underpaid labor
Characterization of platform	An app	A start-up, a disruptor	A traditional capitalist firm, with a tech veneer
Employment status	n/a, formality	Self-employment	Hidden employment/worker status
Implications for worker obligations	No/minimal obligations	Return based on investment	Traditional employment obligations Minimal obligations for minimal pay
Implications for platform obligations	No/minimal obligations	No/minimal obligations	Approaching those of a traditional employer, moderated by employee flexibility
Implications for logic of exchange	Determined by rules of the game/algorithm	Market logic, Pareto efficiency	Reciprocity; Group gain
Implications for platform-courier relationship	Potential for brand loyalty	Minimal trust and loyalty—market rules	Little trust, loyalty—justified by reference to platform betrayals
Implications for courier relationships with restaurants and customers	Minimal obligations, but in-person interactions suggest other social rules apply	Minimal obligations, but market favors professionalism	Comparison to service workers
Implications for courier relationships with other couriers	Friendly competition	Competition; group gain	Solidarity, cooperation

#### Three Narratives: Platform Work as Leisure, Economic Opportunity, or Collectivist Labor

The first narrative of what delivery couriers do highlights the aspects that make it resemble a sport or videogame. Here the emphasis is on the physical performance of couriers, their speed and statistics. A number of couriers we spoke to described their existing passion for cycling, and how delivery riding is “cool.” Treating riding as a sport rather than as a job encouraged workers' intrinsic motivation to improve their performance. Indeed the data generated by couriers and displayed in the platform apps perhaps encourages this understanding, with metrics recorded on various aspects of each couriers' own performance, and foregrounding images of fit couriers in athletic wear. This is indeed reminiscent of Burawoy's account of “making out” at Allied Steel whereby “game playing” provided the emotional and social basis both for daily survival of tough, monotonous industrial labor whilst at the same time allowing for the constitution of the social basis of consent to economic exploitation (Durand and Stewart, 1998).

A second narrative is of platform work as freelancing, whereby couriers are each proprietors of their own small business, claiming more autonomy and flexibility for themselves than they could achieve through traditional employment.

“I like the way it works, I mean everyone's different—there's people that want to work for a company where they've got a lot of job security, for certain reasons—maybe they've got kids or other responsibilities or, I don't know. But then there're other people who really don't care, like myself, all I'm interested in is getting paid for what I do, and if I don't do it and you don't pay me then fair enough. If I go [on holiday] every year […] it won't bother me in the slightest that I won't be getting paid while I'm there. A lot of people would get bothered about that, because they'd be like “well, I feel entitled to it,” but I don't think you are entitled to it—if you're not doing any work, why should you get paid for it? But I guess people have got different ideas about that.” (UK1)

This understanding emphasizes individual responsibility, with the platform cast as a facilitator of couriers' economic empowerment, providing tools for self-employment. Platforms' obligations to couriers thus begin and end with those of a software provider's obligations to its clients. For their part, couriers have no obligations to the platform and are free to behave as they deem fit when interacting with restaurants, customers and other couriers—those who deliver promptly, avoid canceling orders and shifts and gain good ratings from customers and restaurants will find that their business is more successful than that of others. This perception of economic empowerment, as with the first narrative, is undermined by the rider's dependence upon the platform for economic activity sui generis: obligations are one way (from courier to platform), as is responsibility.

A third narrative woven through almost all respondents' testimonies is that of platform work as precarious labor. This latter is typically perceived by workers within the terms of a collectivist framework, defined by Stephenson and Stewart (2001) as workplace collectivism. Here, we might speak of work*space* collectivism, since of course the essential characteristic of platform gig work is the determinate absence of a common workplace. In the public discourse, this understanding is naturally most represented by proto-unions and protestors, as well as academic observers (see, inter alia; Howcroft and Bergvall-Kåreborn, 2014; Rosenblat and Stark, 2015; Aloisi, 2016; Berg, 2016; De Stefano, 2016; Drahokoupil and Jepsen, 2017). The narrative suggests that couriers are exploited and highlights the ways in which delivery riding resembles other low-paid, manual or service work. Couriers themselves underline the insecurity of their earnings due to fluctuations in demand, courier supply and a lack of guaranteed hours for platforms operating shift systems. For those couriers who are financially dependent on platform work, the lack of sick pay is important and encourages a scarcity mind-set (Shah et al., 2012) and over-work. A lack of clarity regarding platform sanctions (account deactivation or unfavorable access to shifts) further exacerbates feelings of precariousness, with some couriers reporting low confidence that they will be permitted to continue working through the platform from 1 week to the next. The couriers generally recognize that they bear a much greater burden of risk regarding fluctuations in the matching of supply and demand than in traditional employment situations, and that there is little cost to the platform of over-recruiting couriers. This precarity is one of the drivers of protests across European countries and the emergence of a collectivist labor narrative (Tassinari and Maccarrone, 2019).

Through our own analysis of the protest materials, we were able to identify specific demands and rhetoric linked to the precarious labor narrative. Despite some variation between countries—potentially attributable to differing legal contexts and thus policies on the parts of the platforms—we found a high level of consistency. Table 3 summarizes the results with percentages and coloring to indicate high and low priorities (green and red, respectively), weighted according to the overall number of references for each country.

**Table 3:** Analysis of protest materials.

	**UK**	**France**	**Germany**	**Italy**
Fair pay	50	48	38	26
Clear, open communication	7	17	22	19
Physical risk burden	8	3	26	17
Job security	8	17	8	12
Respect	8	10	4	11
Holiday and sick pay	6	2	4	7
Loyalty	7	0	0	1
Working conditions—difficulty	1	2	0	4
Working conditions—breaks	4	0	0	0
Promotion opportunities	0	0	0	1
Training	0	0	0	0
Help with personal problems	0	0	0	0
N=	14	10	4	10

*Figures indicate percentage of demands falling into each category (rounded to nearest integer). With shading indicating highest (green) to lowest (red) priorities*.

This analysis suggests that platform workers were not necessarily asking for promotion opportunities, the company to invest in their professional development, significant changes to the nature of the work or recognition of their individual talents and needs. Two high-priority elements—fair pay and job security—might appear to amount to demands for a relational exchange. However, by reviewing each of these references and the coding more carefully, we found that, again, these demands were notably transactional. For example, when it came to demands regarding job security, the protestors call for guaranteed hours and management of labor supply, not long-term contracts. Similarly, their fair pay demands centered on pay for hours actually worked and metric pay, rather than “regular” salaries.

Although the terms and conditions for platform work appear on the surface to be clear, the protest materials reveal evidence of broken promises. We found evidence of poorer conditions imposed unilaterally by the platforms, suggesting, perhaps, that the original or advertised conditions were acceptable to at least some of those who had taken the job to begin with. Secondly, the protestors indicated that the exchange between the worker and the platform requires balance, with many complaints rooted in the imbalance of this power relationship. At the same time, the collective protests also referred to the lack of alternatives as a reason for their disempowerment, explaining why riders enter into and remain in jobs with conditions they considered unacceptable. Furthermore, there is evidence that protestors contest their employment status, highlighting the ways in which they are more akin to employees than self-employed workers and arguing that this classification constitutes a legal loophole exploited by platforms to deprive workers of employment protections and the minimum wage. Overall, couriers registered dissatisfaction with their identity as self-employed workers rather than employees.

A collectivist labor narrative is arguably the driver of a collectivism that many feel has become less present in post-industrial working environments. Not only less present but also structurally less possible due to the apparent fragmentation of the collective worker, a situation reflected in platform organization and labor processes. For example, while some of our respondents claimed that platforms manage rider supply with shifts “to make it a bit fair for everyone” (UK1), others were less trusting of the platforms' intentions, arguing that initial offers of high incentives that were subsequently reduced over time were “deceitful” (DE1) and show that “either they were very naive or they were very cynical” (FR1).

Moreover, those interviewees articulating a collectivist worker narrative frequently perceived their place as workers positioned within an exploitative capital-labor relationship that provided the spur to collectivist labor organization. Speaking about his experience as a courier and union organizer with the IWW, UK5 argued that couriers' protest and strike activity was attributable to a reduction in pay at Platform 1:

“I guess there's a couple of things: one, pay has been steadily going down—every few months, they'll update their pay calculations, and they'll be updated downwards. […] But it's always a little bit complicated. So what they've done over the last year is they've increased the range that they'll sort of cover within a delivery, and they've adjusted the pay system so as to pay us more for the longer journeys but to pay us less for the shorter journeys. And most people fear that overall they're seeing their pay go down as a result, and that like right from the start, that was the assumption that that was what they were playing at. The second thing is that, so in [Platform 1] you need to […] book these hour-long shifts, and they're released on a Monday afternoon for the following week. So you book 2 weeks in advance, to get a priority in booking those shifts, you need to have good statistics, basically a rating system for each rider. *And they've adjusted how that works. So basically, they've got more control over when you're working*. And if you book a shift, previously it was enough just to log on to the account as having attended that shift. *Now you need to log on for a certain amount of time. And assuming you're offered orders, you need to accept at least one of them and so on. So again, it reduces the flexibility of riders and especially those who are working for multiple apps, and might log on to [Platform 1] to tick the box*. But take an order from Platform 2, for example, now you're forced to actually take the orders for [Platform 1]. So the way that people had been sort of gaming it, I guess, has been undermined. And that's caused a lot of– it's a breach of the kind of informal contract relationship between the couriers and [Platform 1]. And that's angered a lot of people and put a lot of people in a bad situation.” (emphasis added)

This is interesting for at least two reasons. First, it highlights the way in which platforms “game” the flexibility process central to the functioning of the business itself and of course, a feature of the gig economy sold to workers as great advance over traditional forms of work. Second, it reveals the extent to which platforms are actually attempting to force workers to make themselves flexible only to one firm: again, using the potential scope promised by platforms for worker autonomy as a way to reduce workers' actual autonomy on the labor market. Moreover, the reasons given by firms for changes to pay did not convince:

“[..] they'll fob us off with a reason to increase efficiency […] [W]ith the recent pay changes they said it was to allow us to become more flexible. Give you a pay cut, so you can become more flexible, it doesn't really—nobody, they will always tag on a reason but it never sells to riders.” (UK5)

This concern over reductions to pay and reduced worker choice over employer and working time was the source of worker mobilization that was, interestingly as our interviewee put it, “quite organic” in the sense that strike actions were, “largely […] self-organized [...] perhaps encouraged by the unions and the knowledge of the presence of unions but not necessarily directly by us” (UK5).

If changes to conditions provide a basis for formal and informal mobilization, as important to collectivism is the management of couriers' labor process. Couriers complain that the way in which platforms detail and provide labor suffers from a lack of transparency and moreover that this opacity is a deliberate feature of the management of courier time, work and labor process organization:

“I think transparency is always something that people are asking for both through the unions [organizing], but also just on the individual level. It might be on the level, as simple as if something goes wrong in a delivery, you try and contact rider support. And they just completely fail to even understand the situation. Perhaps they've accused you of doing something wrong, but they won't tell you what you've done wrong or whatever. So it's that aspect of transparency is a really big concern across the board in that sense that there's a there's a black box that we can shout into with no idea what is happening or what is being said or how decisions are made and so on. So that continues to be a big concern.” (UK5)

“I think if you were having a lot of absences and a lot of holidays I think what [Platform 1] would do would be take your repeat shifts away from you. I don't know how it works, cause I've never really known anyone or experienced it myself, but I'm guessing that's the way it would work that they'd take the repeat shifts away from you. maybe at first maybe half your shifts and if you carried on continued doing it then I guess they'd just take all the shifts away from you and then you'd be on a platform where you'd actually have to apply for shifts and get them approved, and nobody's going to do that cause it's just a headache, so you'd just quit of your own accord, cause it wouldn't be worth it.” (UK1)

What is more, in several instances this lack of trust has been the important factor in a number of protests against platforms:

“They protest when their rights are reduced. What happened with [Platform 1] is, in Paris, they were paid by the hour and after they were paid by the delivery. So it reduced their rights and they weren't happy. It's rare… it happens, but they rarely ask for job security or that kind of thing” (FR2).

In summary we can say that few of the respondents exclusively adhered to any single one of these narratives. Rather, they borrowed from each throughout our interactions in examining different aspects of the work, giving rise to tensions and uncertainty.

The economic opportunity narrative is most straightforward in its implications for couriers' employment status, solidly aligning with claims that couriers are self-employed workers. Here participants emphasize that delivery riding allows them to make money without committing to specific hours or taking orders from a superior, adhering to this narrative of individual responsibility and choice. The leisure narrative, on the other hand, calls the entire premise of the employment status debate into question. If delivering is just part of a game, then maybe it is misguided to ask what type of work it is. While none of our participants went so far as to deny that what they do is work, it seems that its similarity to sports and videogames perhaps dilutes the strength of their claim to remuneration comparable to “traditional” work. The implications of the collectivist labor narrative for the couriers' employment status is less clear-cut. Some value the flexibility platform work provides and express doubt that such flexibility would be possible with a more secure contract. Others seem to suggest that some sort of intermediate status between employment and self-employment might be preferable. Unions and protest groups, for their part, argue that it is incorrect to believe flexibility is incompatible with security.

#### Expectations, Obligations, and Logic of Exchange

One implication of the economic opportunity understanding of platform work might be the expectation of a minimal relationship, and thus minimal obligations, between the platform and the courier. This understanding is clear to see in the interviews. Couriers generally underline the lack of a relationship, noting that they do not believe the platform considers their interests and so highlighting the transactional nature of their relationship. Nevertheless, there is still some disappointment that the platform does not acknowledge or reward exceptional performance:

“if you're having like some accident on the road [Platform 2] only care about if you delivered the parcel and that's it” (UK4)

“it doesn't bother me that they don't care, ‘cause I don't necessarily care about them either. The only thing, like – they're interested in making money, I'm interested in making money, and that's all I really see in it” (UK1)

The app-mediated relationship of couriers with the platform also seems to contribute to the perception that few obligations exist between the courier and the platform. This minimal relationship informs the types of perceived obligations couriers understand. Even those couriers most positive about the platform they use were clear that they owed no loyalty to the platform, and claimed that they would switch to a competitor if they were to offer better pay or conditions. Underlying these minimal obligations seems to be a belief that the exchange with the platform is governed by a purely market logic, which we might understand to be part of the economic opportunity narrative. Couriers recognize that they are valued according to the supply of and demand for their labor, pointing to the low barriers to entry for the job, and the weak bargaining position that comes with it. Others recognized phases in platforms' recruitment of couriers, surmising that platforms initially offer high rewards to entice couriers away from competitors with conditions later deteriorating.

Yet other exchange logics can also be found in the couriers' testimonies. Reflecting a logic of reciprocity, some couriers suggest that they are motivated to work particularly hard or provide good service out of a sense of fair exchange with the platform:

“I don't think you should slack off, ‘cause they are paying you, even if it's a small amount they are paying you—it's only fair” (UK1)

Yet, the logic of reciprocity seems to underlie some couriers' beliefs that they are owed more by the platform, such as pay for time spent waiting for orders as well as other payments associated with more traditional employment relationships including greater support from the platform framed in a logic of group gain. Here, riders' present sick pay, for instance, as a benefit not just for riders but also for the platform.

The collectivist labor narrative diverges from the other two in that it takes the status quo to be unjust and in need of change. Thus, while there is little disagreement regarding what workers and platforms actually provide one another, the collectivist labor narrative presents a normative case for more extensive platform obligations to couriers, currently unfulfilled by platforms. As we saw, courier protest groups, the public proponents of a collectivist labor narrative, most usually articulate a collectivist, critical labor movement orientation. Yet they do not generally ask for promotion opportunities, the company to invest in their professional development, or for significant changes to the nature of the work or recognition of their individual talents and needs. With regards to job security, the protestors call for guaranteed hours and management of labor supply, not long-term contracts. Similarly, their fair pay demands center around increased pay for deliveries realized and time spent on the job, whether waiting for orders or dealing with admin, rather than salaries.

Perceptions of platforms' failures to fulfill their obligations to couriers lead some to protest and strike, while others exit with little noise. In response to platform 1 failing to meet what he perceived to be their obligations, FR1 argued that they “had acted in a very disloyal way and had made it clear that they didn't value me as an employee,” and recounted his use of a number of strategies to make the job easier for himself while artificially improving his metrics. These strategies included using GPS software to fake his location, placing him closer to busy restaurants in the eyes of the platform, and exploiting scheduling bugs so as to get paid for time in which he would not actually have to make deliveries. By way of justification, the rider noted “they'd broken a sort of contract of honesty and reasonable behavior between us, so I no longer felt bound by that” (FR1). In a similar vein, other participants reported their knowledge of riders who, for instance, illicitly make themselves available for work on multiple platforms simultaneously “[be] cause not one of the platforms are guaranteed for you” (UK4). Thus, recalcitrance, cutting corners and reframing, all typical of traditional industrial labor processes, were quite widespread.

## Concluding Discussion

We can understand these exchanges in the platform economy through the lens of the three narratives. The economic opportunity narrative might emphasize couriers' strategic cultivation of a professional reputation and relationships with restaurants and customers, while the leisure narrative might understand interactions with all parties primarily through the rules of the game as established by the platform app. Neither narrative is incompatible with accepting the existence of other incentives for extra work, since the courier is free to develop relationships and motivations external to the exchange between the courier and platform. Nor should they discount the possibility that workers articulating the first and second narratives act in neo-collectivist ways. Their evident workday and workspace reciprocity is a feature of their common sociality: helping one another out with problems, banal or otherwise. The collectivist labor narrative, by contrast, is suspicious of such non-remunerated labor. This extra discretionary effort undertaken for restaurants and customers is crucial for platforms' success, as noted by Rosenblat and Stark (2015) in the case of Uber taxi drivers.

The plausibility of these competing narratives is rooted to some degree in the ways delivery riding presents characteristics of both employment and self-employment. Yet it is also revealing of two critical features of the sociology of digital capitalism of which platform working constitutes a vital part. First and from a worker's point of view, is the evident lack of a clear line of demarcation between employer control and market dictates, especially as platform algorithms become increasingly adept at reflecting and predicting market demand. Given that a workers' employment status is determined partially by their degree of freedom in choosing when and how to work, this ambivalence suggests there is no easy answer to the employment status debate. Indeed in many jurisdictions courts are increasingly recognizing gig economy employees as workers sui generis. Moreover, as one of our interviewees pointed out, riders are wise to ways in which platforms use the “black box” to reduce worker autonomy and thus self-management. In critical labor process theory, the fight over labor control qua the frontier of control is an essential marker of social conflict over the effort-reward bargain. That said, while employment status remains unresolved, conflicting narratives would not unreasonably continue.

Second, what platform working indicates, perhaps surprisingly in forms as acute as traditional industrial work, is the persistence of myriad forms of collectivism. This supports an argument made elsewhere by Stephenson and Stewart (2001), McBride and Martínez Lucio (2011), and Tassinari and Maccarrone (2019) that the focus upon conflict as a marker of collectivism underplays its structural character, which is axiomatic to the capitalist labor process in the form of the collective worker. For that very same reason, therefore an absence of conflict is not reflective of an absence of collectivism: the former is not an ontological marker of the latter. This echoes Stephenson and Stewart's evocation of a “whispering shadow”: even when collectivist behavior appears not to be evident, nevertheless it can be argued that distinctive forms of individualism are themselves inherently linked to collectivism *qua* the collective worker. The collective worker was for Marx about sociology and economics, not space, per se. This is another way of saying that what orientates workers insofar as here we are discussing individualism-collectivism, is not *where* people work so much as how and for whom (their common employer) and under what conditions. Thus, arguably the individualistic narratives of economic opportunity and leisure, especially, echo a pattern of coping with the stress of the platform labor process in ways reminiscent of Burawoy's factory workers game playing (“making out”) strategies (see above Table 2 “Leisure”). While research on workplace collectivism in traditional industrial cultures focuses upon the immediacy of working together in the same geographical space, it may be too readily assumed that commonality of space is *the key* determinant of common experiences which in turn allows for (trade union) collectivist action. Yet one of the factors allowing for collectivism amongst gig workers is precisely the attribute that leads to geographical fragmentation—the device itself. Moreover, and this is one of the paradoxes of the platform economy labor process, it is exactly the courier's ability to utilize the technologies of the platforms, including gaming the app, that allows fellow workers delivering several kilometers apart to share information just as immediately as two automotive workers in the same factory. The very technology that divides the collective worker spatially can also provide mechanism that brings them together socially: moreover, bringing people together socio-economically into a ‘common' working context by virtue of exploitation by the same employer in itself does not lead to collective action that is always conflictual.

Given this insight, we argue that early perspectives dating back two decades on the meaning of collectivism and the collective worker, Beck (2000) being perhaps the most pessimistic, tended toward the view that since factory workers in industrial capitalism constituted the archetypal collectivist actor, their demise spells the end of collectivism as such and solidaristic trade unionism in particular. Yet as our research and that of others suggests, new forms of labor and labor exploitation belie the idea that individualist, neoliberal narratives spell of the end of collectivism never mind the collective worker (Woodcock and Graham, *op cit*). On the contrary, both over-work and actor commitment to *plus* working serve to underscore the reality that a deindustrializing economy is certainly not leading to a decollectivising society. Neither is it an economy in which forms of trade unionism have become less salient. In this respect, we can recall Silver's point to the effect that we may be witnessing the growth, however febrile, of a collectivist trade union agenda amongst workers.

## Data Availability Statement

The datasets generated for this study are available on request to the corresponding author.

## Author Contributions

All authors listed have made a substantial, direct and intellectual contribution to the work, and approved it for publication.

## Conflict of Interest

The authors declare that the research was conducted in the absence of any commercial or financial relationships that could be construed as a potential conflict of interest.

## Footnotes

^1^Huws in particular unpacks the functionalist rhetoric of the soi-disant sharing economy – viz, “it works to everyone's benefit so it must be positively beneficial” - highlighting the extent of capture by capital of what was indeed originally a form of social exchange within private sharing spaces outside the regulatory norms attending the commodification of our private lives and common spaces.
